# Immobilized Enzymes on Magnetic Beads for Separate Mass Spectrometric Investigation of Human Phase II Metabolite Classes

**DOI:** 10.1021/acs.analchem.3c02988

**Published:** 2023-08-08

**Authors:** Ioanna Tsiara, Amelie Riemer, Mario S. P. Correia, Ana Rodriguez-Mateos, Daniel Globisch

**Affiliations:** § Department of Chemistry - BMC, Science for Life Laboratory, Uppsala University, 75124 Uppsala, Sweden; † Department of Nutritional Sciences, School of Life Course and Population Sciences, Faculty of Life Sciences and Medicine, King’s College London, London SE1 9NH, UK

## Abstract

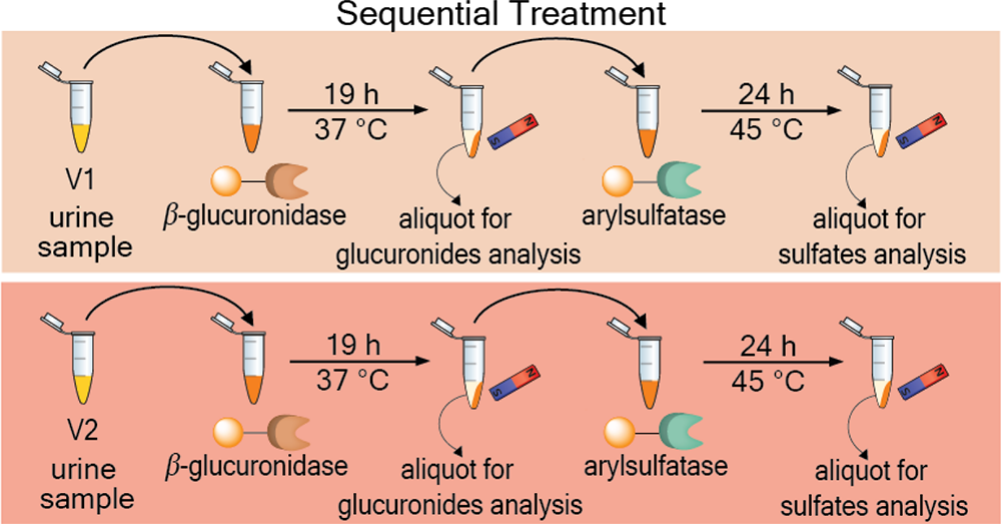

The human body has evolved to remove xenobiotics through a multistep clearance process. Non-endogenous metabolites are converted through a series of phase I and different phase II enzymes into compounds with higher hydrophilicity. These compounds are important for diverse research fields such as toxicology, nutrition, biomarker discovery, doping control, and microbiome metabolism. One of the challenges in these research fields has been the investigation of the two major phase II modifications, sulfation and glucuronidation, and the corresponding unconjugated aglycon independently. We have now developed a new methodology utilizing an immobilized arylsulfatase and an immobilized β-glucuronidase to magnetic beads for treatment of human urine samples. The enzyme activities remained the same compared to the enzyme in solution. The separate mass spectrometric investigation of each metabolite class in a single sample was successfully applied to obtain the dietary glucuronidation and sulfation profile of 116 compounds. Our new chemical biology strategy provides a new tool for the investigation of metabolites in biological samples with the potential for broad-scale application in metabolomics, nutrition, and microbiome studies.

## Introduction

The detoxification and clearance process in the human body are considered to be emerging fields for the investigation of microbiota-derived and diet-derived metabolites.^1−3^ Detoxification is a central process in metabolism that facilitates the removal of xenobiotics and endogenous compounds, carried out in a biphasic process.^4^ Xenobiotics, also known as exogenous compounds, comprise all chemicals that organisms cannot produce but to which an organism is exposed throughout their lifetime, such as dioxins, food additives, and pharmaceuticals.^5,6^ These chemical substances are absorbed by the human body through environmental exposures, nutrition, drug administration, and lifestyle choices.^7,8^ Additionally, most metabolites produced by the gut microbiota are xenobiotics as well and are cleared through the same process as they can have toxic effects on the human host.^9^ The importance of the microbiome metabolism on the conversion of dietary compounds has been revealed to impact human physiology.

The first modification comprises oxidation, reduction, or hydrolysis to insert functional groups such as alcohols and amines for subsequent biotransformation (phase I modification). Cytochrome P450 oxidases are the leading enzymes in the first modification step that add polar groups to xenobiotics to increase the metabolite hydrophilicity. This is followed by conjugation reactions including glucuronidation, sulfation, methylation, acetylation, and amino acid conjugation that are characterized as phase II modifications. The two major phase II modifications in humans are glucuronidation and sulfation (40–55 and 25–35%, respectively).^4,10^ The key enzymes that catalyze glucuronidation reactions of various xenobiotics and endogenous compounds are uridine–diphosphate–glucuronosyltransferases (UGTs). During these biotransformation reactions, a glucuronic acid moiety is conjugated via UGTs for covalent modification of substrates containing a nucleophilic functional group such as alcohols, amines, and carboxylic acids.^11^ Sulfated metabolites have been identified as key regulators of microbiota–host interactions.^12^ The investigation of the human sulfatome is therefore of great importance for the elucidation of these metabolic exchanges.^13,14^

The enzymatic hydrolysis of sulfated and glucuronidated metabolites is the most common method for their investigation and discovery.^14^ β-Glucuronidases and sulfatases reverse the natural conjugation reaction and have been applied for diverse sample types. For advanced analysis of these compound classes, we have recently developed a series of enzymatic sample pre-treatment methods for analysis of sulfated metabolites (sulfatome analysis) and glucuronidated metabolites. The main enzymes used for the investigation of known and unknown metabolites were from the snail *Helix pomatia*.^15,16^ However, this commercially available enzyme is of high impurity and contains a mixture of enzymes with several bioactivities with the glucuronidase activity as the highest.^17^ To utilize pure enzymes for selective analysis of a single compound class, the enzymes were either purified or recombinant enzymes have been introduced.^17−19^

While a one-time use of enzymes has been established in most analytical settings for standard investigations, new methods for a more efficient analysis and optimized use of material are required. The immobilization of enzymes to various types of solid support has been described to achieve better specific activities and selectivity.^20,21^ Magnetic beads have been used in mass spectrometric methods for affinity capturing of proteins or ligands.^22,23^

**Figure 1:**
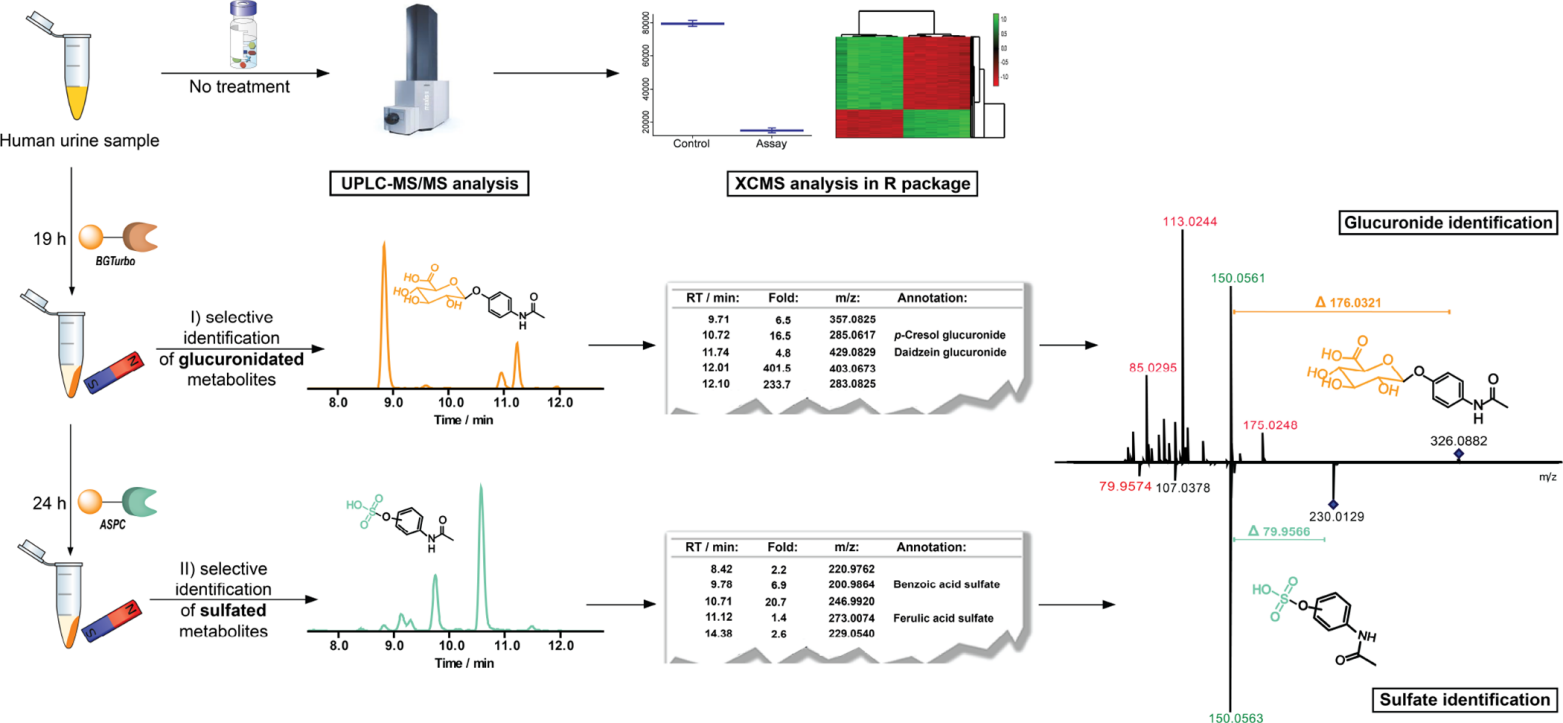
Workflow of our methodology illustrating the identification and structure validation of acetamidophenol glucuronide and acetamidophenol sulfate.

In this study, we sought to develop a new and simple enzymatic assay through immobilization of the recombinant β-glucuronidase BGTurbo and the recombinant arylsulfatase ASPC to magnetic beads for the first sequential investigation of phase II modifications (Figure 1). This methodology allows for high reusability of the enzyme, robustness, separate analysis, and simple sample handling. This experimental setup allows for the first time to separately analyze xenobiotic metabolites as unconjugated, sulfated, and glucuronidated compounds. Sample analyses of a dietary intervention study using optimized immobilization and treatment conditions allowed for the investigation of 52 glucuronidated and 64 sulfated metabolites including several microbiome-derived metabolites. Our established method can easily be modified for application of other enzymes to investigate metabolite classes of interest and is thus applicable for all bioanalytical applications.

## Experimental Section

### Ethical Approval

The study was conducted in accordance to the guidelines stated in the current revision of the Declaration of Helsinki, and informed consent was obtained for all subjects. All procedures involving human samples were approved by King’s College London Research Ethics Committee (HR-17/18–5353) and registered at the National Institutes of Health clinicaltrials.gov as NCT03573414.

### Glucuronidase Assay

A mixture of six glucuronidated compounds (*N*-acetyltyramine-*O*,β-glucuronide (**1**), phenyl-β-glucuronide (**2**), *p*-nitrophenyl-β-d-glucuronide (**3**), *p*-acetamidophenyl-β-d-glucuronide (**4**), estrone-β-d-glucuronide (**5**), and 4-methylumbelliferyl-β**-**d-glucuronide (**6**)) was prepared (500 μM each in MQ-water). Glucuronidase activity was determined based on a protocol (Supporting Information).^19^ In every assay, 100 U of BGTurbo glycerol free-high efficiency recombinant β-glucuronidase (Kura Biotech, cat no. BGTgf-5 mL, lot no. 7623) was used. Aliquots were collected at five time points (0, 0.5, 1, 4, and 24 h); the enzyme was precipitated using methanol to quench the enzymatic reaction. The supernatant was collected after centrifugation (5 min, 14,100*g*) and dried under vacuum in a Speedvac. Samples were reconstituted in 5% acetonitrile in water prior to UHPLC–MS analysis.

### Sulfatase Assay

A mixture of six sulfated compounds (methylurolithin sulfate (**7**), *p*-cresyl sulfate (**8**), *N*-acetylserotonin sulfate (**9**), estrone-3-sulfate (**10**), 4-ethylphenyl sulfate (**11**), and indoxyl sulfate (**12**)) was prepared (500 μM each in 50 mM ammonium acetate). Sulfatase activity was determined based on a protocol (Supporting Information).^17^ In every assay, 5 U of the ASPC recombinant arylsulfatase (KuraBiotech, cat no. ASPC-10 mL, lot no. 6604-1) was used. Aliquots were collected at five time points (0, 0.5, 1, 4, and 24 h); the enzyme was precipitated using methanol to quench the enzymatic reaction. The supernatant was collected after centrifugation (5 min, 14,100*g*) and dried under vacuum in a Speedvac. Samples were reconstituted in 5% acetonitrile in water prior to UHPLC–MS analysis.

### Immobilization to Magnetic Beads

MagnaBind carboxyl-derivatized bead slurry (100 μL, Thermo Fisher Scientific) was transferred into a 1.5 mL Eppendorf tube. The beads were washed twice with 100 μL of 25 mM MES (*N*-morpholinoethane sulfonic acid) buffer for 5 min with thorough shaking (Thermomixer, 25 °C, 1100 rpm). The magnetic beads were then activated by addition of 50 μL of EDC [1-ethyl-3-(3-dimethylaminopropyl)carbodiimide hydrochloride], 50 μL of NHS (*N*-hydroxysulfosuccinimide), and 50 mg/mL in cold 25 mM MES pH = 5 and dissolved immediately before each use. The solution was mixed and incubated with tilt rotation at room temperature for 30 min (Thermomixer, 25 °C, 400 rpm). After incubation, the Eppendorf tube with the mixture was placed on the magnet for 4 min and the supernatant was removed. The beads were then washed twice with the same MES solution, pH = 5 (Thermomixer, 25 °C, 400 rpm). After the activation, aliquots of the β-glucuronidase BGTurbo (100 U) and arylsulfatase ASPC (5 U) were added separately to the activated MagnaBind beads for the enzymatic treatment of the urine samples. For the negative control sample, only the beads with buffer were used. The solutions were incubated overnight with tilt rotation at room temperature (Thermomixer, 25 °C, 300 rpm). After the incubation, the tubes were placed on the magnet for 4 min and the supernatant was removed. The coated MagnaBind beads were washed as described above before the urine sample was added.

### Urine Sample Preparation

Exactly 10 μL from 10 different urine samples was pooled to constitute 100 μL for each time point of urine collection (V1 and V2 separately; Table S1). Ice cold methanol (400 μL) was added to each of the urine samples for protein precipitation. The samples were vigorously shaken for 30 s and then cooled at 4 °C for 30 min. Upon protein precipitation and centrifugation at 14,100*g* for 5 min, the extracted urine samples were dried in vacuo at ambient temperature. The residues of the tubes were dissolved in 100 μL of Instant Buffer I (Kura Biotech, lot no. 2319).

### Sequential Enzymatic Treatment

The reconstituted pooled urine sample was added to the magnetic beads coupled with BGTurbo (100 U). After a 19 h incubation with the β-glucuronidase, the beads were placed on the magnet and 25 μL of the solution was removed for the analysis of the glucuronidated metabolites. The remaining supernatant was transferred for the arylsulfatase treatment to the beads coupled to ASPC, and after 24 h of incubation, the beads were placed on the magnet and 25 μL of the solution was removed for the analysis of the sulfated metabolites. The same process was performed for both V1 and V2 time points, as well as for the negative control sample, for which aliquots were collected at time points of 0 and 24 h. Ice cold methanol (100 μL) was added to each of the 25 μL aliquots. After centrifugation (14,100*g* for 5 min), the supernatant was collected and dried in vacuo. Afterward, the remaining pellet was dissolved in 50 μL of water/acetonitrile (95/5, v/v). The supernatants were then transferred to LC vials for the UHPLC–MS/MS analysis. Each sample was injected four times using a randomized sequence of control and assay samples to avoid biased results.

### LC–MS Analysis

The UHPLC–MS/MS analysis was performed in a Maxis II ETD Q-TOF mass spectrometer (Bruker Daltonics, Germany) using an electrospray ionization (ESI) source with either an Elute UHPLC (Bruker Daltonics, Germany) or a 1260 Infinity II Binary Pump (Agilent Technologies, USA) system. The separation was performed on an Acquity UPLC HSS T3 column (1.8 μm, 100 × 2.1 mm) from Waters Corporation. Milli-Q water with 0.1% formic acid was used as mobile phase A, and LC–MS grade methanol with 0.1% formic acid was used as mobile phase B. The column temperature was kept at 40 °C, and the autosampler temperature was kept at 4 °C. The flow rate was set to 0.22 mL/min with an injection volume of 5 μL. The gradient used was as follows: 0–2 min, 0% B; 2–15 min, 0–100% B; 15–16 min, 100% B; 16–17 min, 100–0% B; 17–23 min, 0% B. The system was controlled using the Compass HyStar software package from Bruker (Bruker Daltonics, Germany). High-resolution mass spectra were acquired in negative mode at a mass range of *m*/*z* 50–1200. Data acquisition was performed in AutoMSMS mode (data-dependent acquisition, DDA) with a cycle time of 0.5 s and a ramped collision energy from 20 to 50 eV. A solution of sodium formate [10 mM in a mixture of 2-propanol/water (1/1, v/v)] was used for internal calibration at the beginning of each run, in a segment between 0.10 and 0.31 min.

### Data Analysis

Data analysis was performed using the XCMS metabolomics software package under R (version 4.2.1), using a script designed to identify features with a *m*/*z* difference of 79.9568 Da for sulfated metabolites and 176.0321 Da for glucuronidated metabolites.^24−26^ The data was processed, and hits containing a glucuronic acid moiety, a sulfate ester, or their corresponding aglycons were identified using the following criteria: 1.2-fold change for the control group, an intensity level higher than a 15,000 ion count, and 10 ppm mass accuracy. Hydrolysis curves and bar charts were generated using GraphPad Prism 9.0 software (GraphPad Inc., San Diego, CA).

## Results and Discussion

Phase II modifications are of increased importance for the investigation of diet-derived compounds. Especially, dietary metabolites produced or converted by the microbiome can either be beneficial or toxic to the human host. Several mass spectrometric assays have been utilized so far; however, more advanced and selective methods are needed to investigate known metabolites as well as identify yet unknown metabolites. It is a crucial step to first identify these compounds present in the human body, elucidate their chemical structure, and determine their bioactivity.

**Figure 2:**
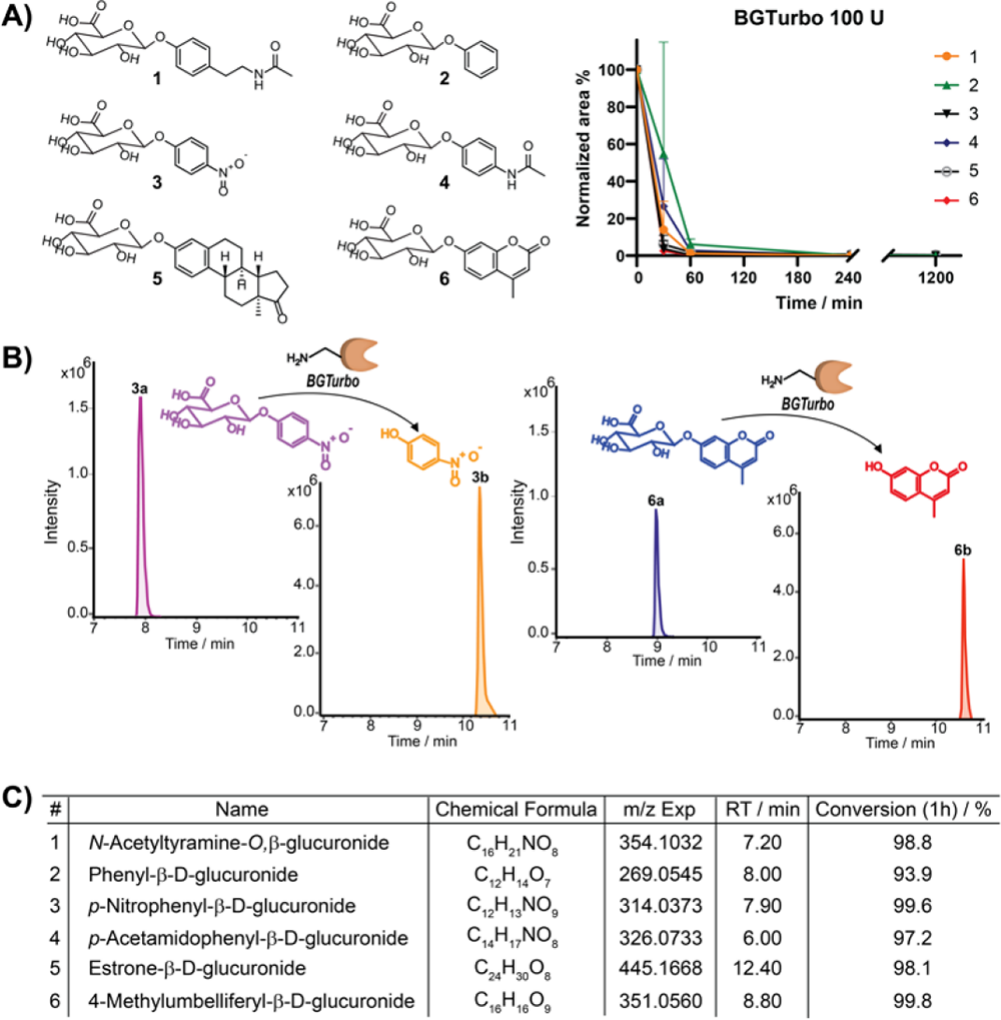
(A) Structures of glucuronidated metabolites (**1**–**6**) tested as substrates in the enzymatic assay (left). Hydrolysis experiments of **1**–**6** treated with the β-glucuronidase BGTurbo (100 U) performed in triplicate (right/error bars: SD). (B) Representative extracted ion chromatograms (EICs) for the enzymatic hydrolysis of *p*-nitrophenyl-β-d-glucuronide (**3a/***m*/*z* = 314.0512) and 4-methylumbelliferyl-β-d-glucuronide (**6a**/*m*/*z***=** 351.0722) to *p*-nitrophenol (**3b/***m*/*z***=** 138.0191) and 4-methylumbelliferone (**6b/***m*/*z***=** 175.0395), respectively. (C) Table with the selected glucuronidated metabolites and their conversion rate after 1 h of treatment with BGTurbo.

Our envisioned analysis requires enzymes that are stable, efficient, and promiscuous for hydrolysis of the specific compound class. The recombinant β-glucuronidase of our choice is BGTurbo and the arylsulfatase ASPC. We have recently utilized and evaluated the latter enzyme for broad-scale analysis of biological sulfated metabolites.^18^ Prior to the analysis, we have first investigated the hydrolysis efficiency of BGTurbo for six glucuronidated metabolites of diverse structure: *N*-acetyltyramine-*O*,β-glucuronide (NATOG, **1**), phenyl-β-glucuronide (**2**), *p*-nitrophenyl-β-d-glucuronide (**3**), *p*-acetamidophenyl-β-d-glucuronide (**4**), estrone-β-d-glucuronide (**5**), and 4-methylumbelliferyl-β-d-glucuronide (**6**). To identify the optimum enzyme concentration for these substrates, we screened five different BGTurbo units (0.1, 1, 10, 50, and 100 U). We observed fast hydrolysis of at least 94% after 1 h of incubation for all six substrates with 100 U of BGTurbo. Due to the nearly complete hydrolysis of all six substrates after 1 h with 100 U, we decided to continue with this enzymatic activity for all the following analyses (Figure 2A). The chromatographic separation of all glucuronidated metabolites and corresponding aglycons is shown in Figure 2B,C. These six compounds with a broad range of physical properties build the foundation for the analysis of human samples.

**Figure 3:**
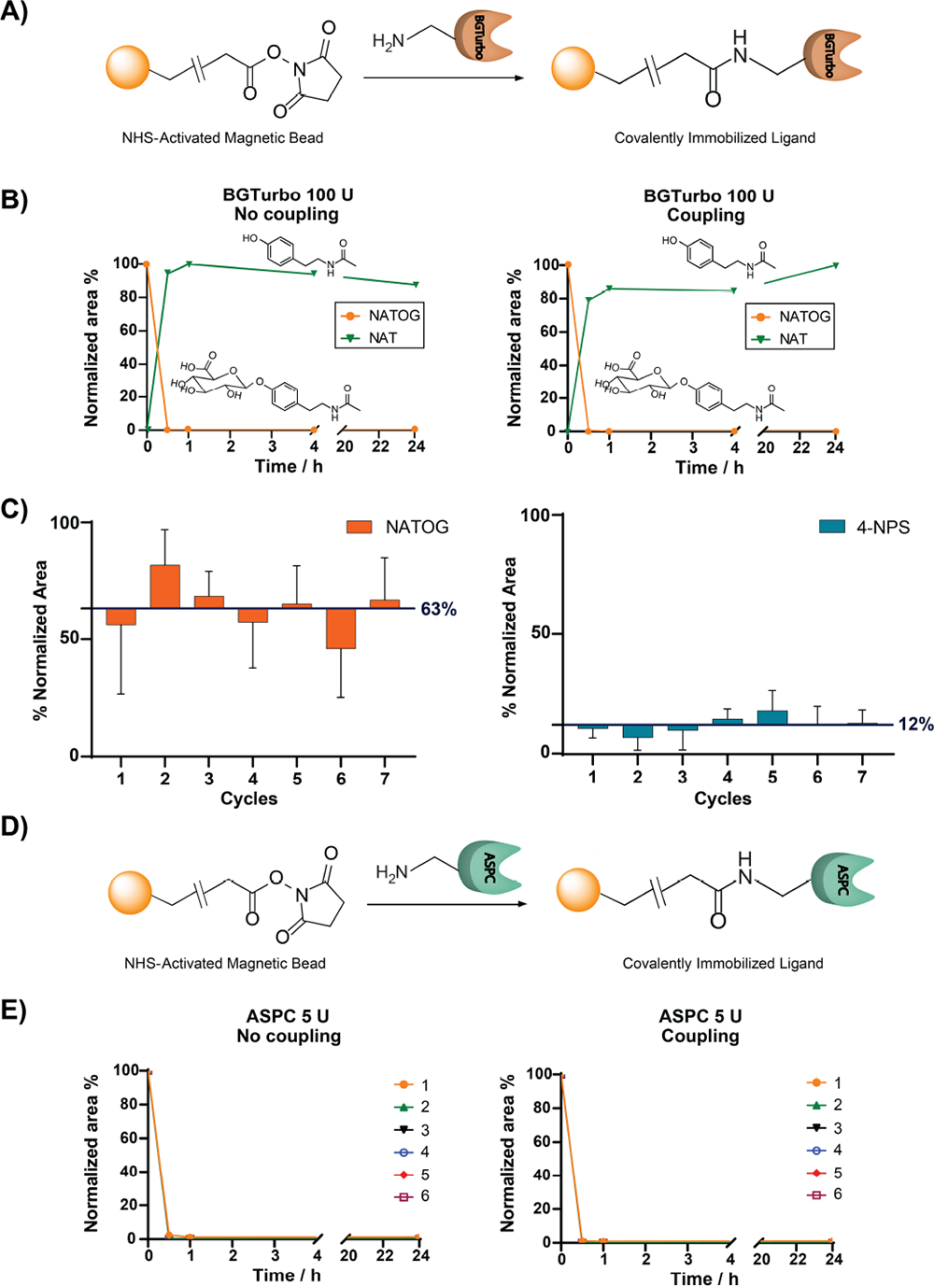
(A) Coupling of β-glucuronidase BGTurbo using NHS-activated magnetic beads. (B) Hydrolysis curves for NATOG and formation of the aglycon *N*-acetyltyramine (NAT) using uncoupled (left) and coupled BGTurbo (right). (C) Recycling experiments for the immobilized β-glucuronidase (left) and arylsulfatase (right) to beads for seven cycles and treated for 1 h, each performed in triplicate (error bars: SD). The line at 63% indicates the overall mean of the % conversion of NATOG in the seven cycles. The line at 12% indicates the overall mean of the % conversion of 4-nitrophenyl sulfate (4-NPS) in the seven cycles. (D) Coupling of arylsulfatase ASPC using NHS-activated magnetic beads. (E) Hydrolysis experiments of selected sulfated metabolites using uncoupled (left) and coupled ASPC (right).

As our methodology required immobilized enzymes, we have first evaluated the stability of BGTurbo after immobilization to the magnetic beads. The coupling procedure followed standard peptide coupling conditions using NHS activation of MagnaBind carboxyl-derivatized beads (Figure 3A). The activity after immobilization was determined with the standard activity protocol, and 100 U of the active enzyme was used for the hydrolysis experiments of the substrate NATOG.^6,27,28^ Importantly, the hydrolysis curves for the immobilized enzyme versus enzyme in solution were highly similar, which demonstrates that the enzyme structure is undistorted (Figure 3B). With these successful initial results, we determined the reproducibility of the same coupled β-glucuronidase for hydrolysis of NATOG (591 μM) at 1 h. The reusability of the immobilized β-glucuronidase was investigated for seven cycles in triplicate, and the results demonstrated sufficient reproducibility and thus the versatility of the immobilized enzyme (Figure 3C, left). We have also determined the carry-over by designing an experiment with five different compounds (>500 μM) that were sequentially treated with the same immobilized β-glucuronidase. The carry-over determined for the aglycon and glucuronide was in all cases less than 0.8% (Figure S1). This is also strong evidence for a low corona formation of the metabolites onto the magnetic beads that has been reported for other applications (Table S2).^29,30^ We have now determined the recovery to be between 73 and 87% for sulfated and glucuronidated metabolites, respectively.

We have next obtained the hydrolysis profiles of a mixture of six standard compounds in comparison to the uncoupled enzyme (Figure S2). The results confirmed that the β-glucuronidase after coupling to magnetic beads remains highly active with rapid hydrolysis of all six glucuronidated compounds reaching a minimum of 80% conversion after 1 h of incubation. This shows that the enzyme maintains its major activity after coupling to the magnetic beads that is required for our method and proves an intact active site of BGTurbo.

For investigation of sulfated metabolites, we have utilized the arylsulfatase ASPC. We have previously investigated this enzyme for promiscuous substrate activity in human samples.^18^ In here, we have immobilized ASPC to magnetic beads using the same immobilization conditions as for BGTurbo (Figure 3D). The hydrolysis profile of a mixture of six selected sulfated metabolites of diverse structures with ASPC (5 U) in solution compared to the immobilized arylsulfatase was identical with rapid hydrolysis within the first 30 min (Figure 3E). We also tested the option for a repeated analysis with the same immobilized arylsulfatase, and the seven cycles demonstrated high reproducibility of substrate hydrolysis (Figure 3C, right). The reproducible results for both enzymes are important for the application in human samples.

**Figure 4:**
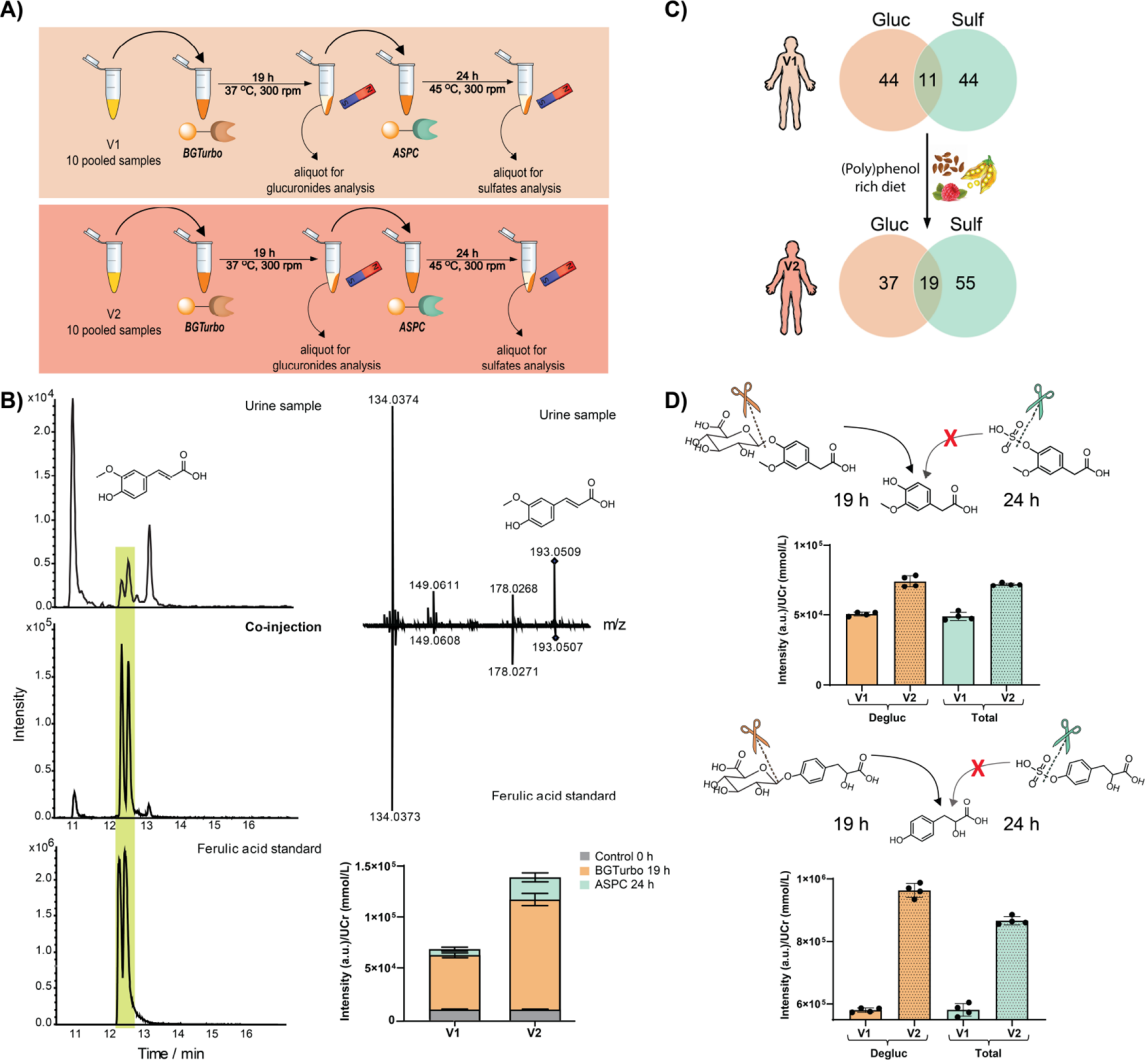
(A) Experimental workflow for treatment of human urine samples before (V1) and after (V2) dietary intervention using our developed methodology. (B) Identification of ferulic acid by co-injection experiments with an authentic reference standard (extracted ion chromatograms, *m*/*z* = 193.0501). The bar graphs illustrate the formation of ferulic acid after 19 h of treatment with BGTurbo (orange) followed by 24 h of treatment with ASPC (green) comparing V1 and V2. The amount of the initial ferulic acid present in the control sample is illustrated with gray color. (C) Venn diagram that shows the number of glucuronidated (orange, Gluc) and sulfated (green, Sulf) metabolites identified after the enzymatic treatment of human urine samples. The overlap represents the number of metabolites that were both sulfated and glucuronidated in the same human sample as well as numerical differences before (V1) and after dietary intervention (V2). (D) Investigation of the regioisomers homovanillic acid and hydroxyphenyllactic acid in V1 and V2 after treatment with the BGTurbo (orange) and the ASPC (blue). “Degluc” represents the intensity of the aglycon after the β*-*glucuronidase treatment, and “Total” represents the final intensity of the aglycon after the sequential β*-*glucuronidase and arylsulfatase treatment. Error bars: SD of the four UHPLC–MS injections.

In the next step, we applied our new methodology to human urine samples collected in a dietary intervention study. The samples were collected from individuals before (V1) and after (V2) the consumption of a (poly)phenol-rich diet as described for our sulfatome analysis.^14^ To test the feasibility of a sequential analysis with both enzymes to analyze both metabolite classes within a single sample, we split the sample in two parts. In the first experiment, the samples were first treated with β-glucuronidase and then with arylsulfatase, whereas the reverse order was applied for the second experiment (Figure S3). The results from the two individual experiments revealed that BGTurbo’s activity is negatively influenced by the buffer used for ASPC evident through a lower number of discovered glucuronidated features after the selective XCMS analysis. The activity of the arylsulfatase ASPC was nearly unaffected by the change of buffer. Thus, we decided that the experimental setup for human samples is a first treatment with BGTurbo followed by treatment with ASPC (Figure 4A). For this exploratory analysis, we pooled 10 samples separately from the V1 group (before diet) and 10 samples from the V2 group (after diet).

Each pooled sample was first treated with BGTurbo immobilized to magnetic beads for 19 h (Figure 4A). After this incubation, the supernatant was removed upon magnetic separation and transferred to new vials, and aliquots were removed. To the remaining solution, we added ASPC immobilized to magnetic beads for the second round of incubation for 24 h. The solution was removed afterward by magnetic separation. All aliquots were collected after each enzymatic incubation step, and the control samples were analyzed via UHPLC–MS in a randomized sequence. The LC–MS dataset was processed with XCMS as described earlier to specifically identify the sulfated and glucuronidated metabolites.^14,19^ The advantages of this new method is the possibility to investigate different compound classes separately upon specific enzymatic conversion. Glucuronides were identified by comparing the BGTurbo-treated sample with the untreated control sample. Sulfated metabolites were either identified by comparison of the ASPC-treated sample with the untreated control sample or with the BGTurbo-treated sample.

The features were filtered to remove nonspecific signals, and the most altered metabolite structures were identified (fold change >1.2). Using this bioinformatic process, we identified metabolites for each phase II modification that fell within these criteria and were most abundant, for which we determined the chemical structure (confidence level (CL) 1 and 2) or the chemical formula (CL 3). The metabolite structure was either validated via authentic standards (CL 1) or MS/MS fragmentation analysis (CL 2; Tables S4 and S5). In the case of the glucuronidated metabolites and limited available standards, we validated the structures of the aglycon with standards as we had developed earlier (Figure 4B).^19,31^ MS/MS fragmentation experiments were performed for the validation of glucuronidated compounds and their aglycons in comparison with MS/MS libraries such as HMDB and SIRIUS (Figure 4B).^32,33^ After the identification of the metabolite structure for all three metabolite forms (unconjugated, glucuronidated, and sulfated) in the urine samples, we are able to relatively compare the quantities of each metabolite conjugate as well as the aglycon within the sample after normalization to the creatinine levels and control samples.

In total, we identified 11 metabolites in V1 and 19 metabolites in V2 for which we detected both phase II modifications (Figure 4C). This is demonstrated for ferulic acid as one example, a metabolite that can either be derived from food intake or be produced by the gut microbiota (Figure 4B).^34,35^ As expected from our previous study, the metabolite and its modifications were present at higher concentrations in the V2 compared to the V1 samples.^14^ Ferulic acid is derived from the anthocyanin compound class of the combined polyphenolic diet. Interestingly, we can now determine that the majority of the metabolites in the V1 and the V2 sample sets was present as the glucuronidated metabolite, while the corresponding aglycon and its sulfated form were present at lower quantities (Figure 4B). This example demonstrates the powerful potential of our method for future quantification of a metabolite at the aglycon level, more precisely by consideration of the aglycon itself, the glucuronide, and the sulfate forms independently.

This separate semi-quantitative analysis has not been described before as the tools were missing, and all three internal standards were required. We have also performed this separate analysis for the two regioisomers homovanillic acid and hydroxyphenyllactic acid, which represent another bottleneck in metabolomics research to distinguish between structural isomers. Each structure was validated using internal standards for the corresponding aglycons.^19^ Metabolite peak intensities in the different treated samples led to the determination of the conjugate quantities. For both regioisomers, we solely identified the glucuronidated metabolite in the samples, again with higher levels observed in the V2 sample set after dietary intake (Figure 4D). Due to the lack of sulfated metabolites, similar levels of homovanillic acid and hydroxyphenyllactic acid were observed within the sequential treatment for both V1 and V2 with ratios to be at 1:1 and 1.1:1 after β-glucuronidase (Degluc) and arylsulfatase (Total) treatment, respectively.

As absolute quantification of each metabolite and its phase II modification is desirable but depends on the availability of isotope-labeled internal standards, we have performed one example for the quantification of ferulic acid in a different pooled urine sample (Table S3 and Figure S4). This demonstrates the reproducibility of our assay and that the identified metabolites of interest from the broad discovery approach can be further investigated for clinical applications in a related targeted analysis.

## Conclusions

In the present study, we have developed a new methodology for the targeted investigation of glucuronidated and sulfated metabolites in human samples. Immobilization to magnetic beads of two different recombinant enzymes allowed for the separate investigation of both phase II metabolite classes that we have termed conjugatome analysis. The method allows for the simple treatment of human urine samples independently for the separate detection and investigation of glucuronidated and sulfated metabolites from a single sample. This strategy combined with our bioinformatic data analysis and control sample strategy was efficiently applied for the sequential enzymatic treatment and identification of metabolites in human urine samples. We have confirmed upregulated levels of dietary compounds and their modifications after a polyphenolic breakfast in the investigated pooled urine samples. This methodology overcomes the limitation of previous reports where either both compound classes were enzymatically converted together or one single enzyme was used for a targeted analysis of one metabolite class. This chemical biology methodology is a new tool for general application for the selective investigation of phase II metabolite classes in the nutrimetabolomics and microbiome research fields.

## Supporting Information Available

Supporting figures and tables, general methods, study design, determination of glucuronidase activity, determination of arylsulfatase activity, carry-over effect, cycle analyses of the immobilized enzymes, metabolite corona formation, two-cycle experiment, quantification of ferulic acid, and reference (PDF)
