# Supplementary material for: Immobilized Enzymes on Magnetic Beads for Separate Mass Spectrometric Investigation of Human Phase II Metabolite Classes
Source: Anal Chem. 2023 Aug 8;95(33):12565–71. doi: 10.1021/acs.analchem.3c02988 (PMC10456218; doi:10.1021/acs.analchem.3c02988)
Supplement: Supplementary file 1 — ac3c02988_si_001.pdf [file ac3c02988_si_001.pdf]

SUPPORTING INFORMATION

Immobilized Enzymes on Magnetic Beads for Separate Mass Spectrometric Investigation of Human Phase II Metabolite Classes

Ioanna Tsiara,[a] Amelie Riemer,[a] Mario S.P. Correia,[a] Ana Rodriguez Mateos,[b] and Daniel Globisch*[a]

[a] Department of Chemistry - BMC, Science for Life Laboratory, Uppsala University, 75124 Uppsala, Sweden

[b] Department of Nutritional Sciences, School of Life Course and Population Sciences, Faculty of Life Sciences and Medicine, King's College London, UK

Table of contents

Figures 2
Tables 5
General methods........ 13
Study design 13
Description of procedures 14
Determination of glucuronidase activity 14
Determination of arylsulfatase activity........ 15
Carry-over effect 15
Cycle analyses of the immobilized enzymes........ 16
Metabolite corona formation........ 17
Two-cycles experiment 17
Quantification of ferulic acid 18
Reference........ 18

Figures

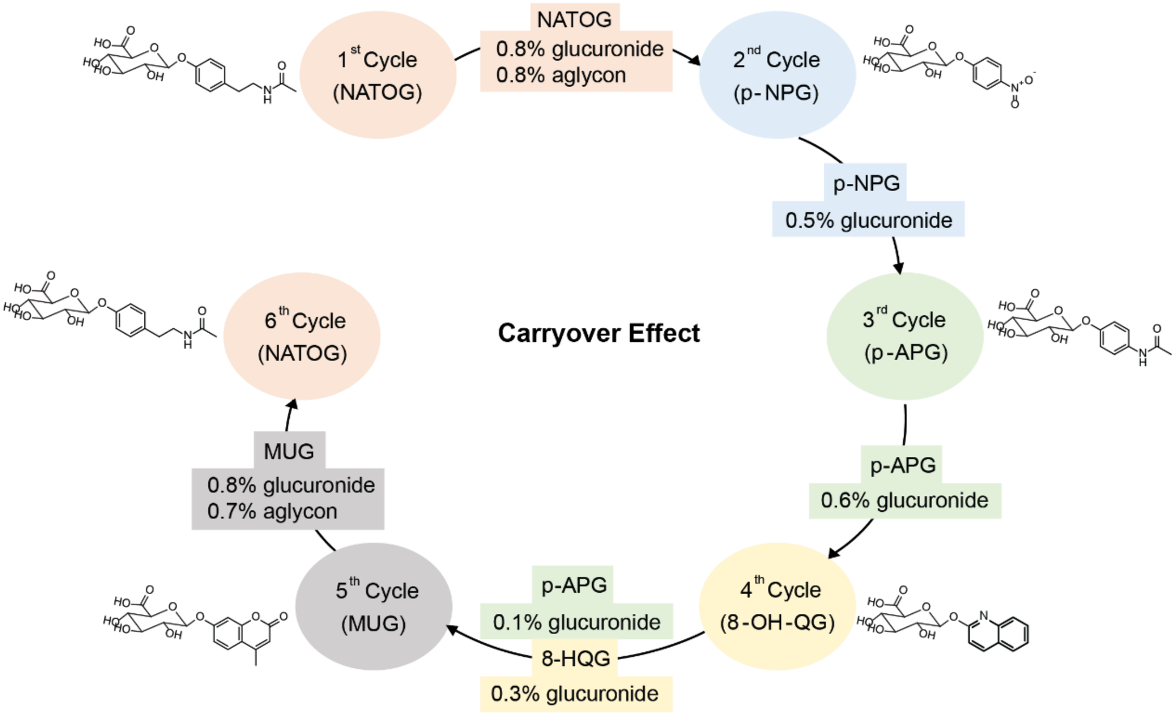

Figure S1. Overview of carry-over experiments. Six cycles of 1 h incubation with 100 U of BGTurbo® coupled to magnetic beads were performed using five different substrates, *N*-acetyltyramine-*O*,*β*-glucuronide (NATOG), *p*-nitrophenyl-*β*-D-glucuronide (*p*-NPG), *p*-acetamidophenyl-*β*-D-glucuronide (*p*-APG), 8-hydroxyquinoline-*β*-D-glucuronide (8-OH-QG), 4-methylumbelliferyl-*β*-D-glucuronide (MUG). The percentage of carry-over was calculated for both the glucuronide and the aglycon within every cycle.

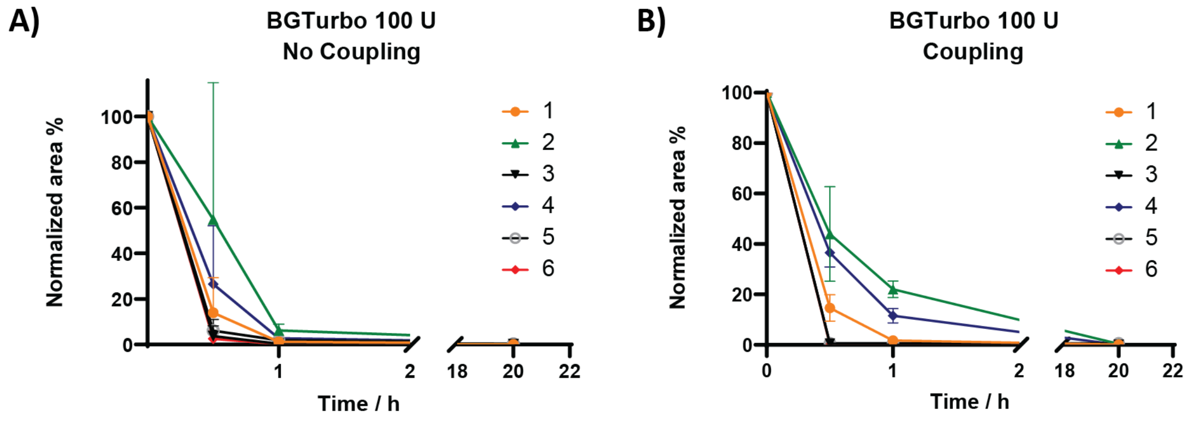

Figure S2. Comparison of hydrolysis curves of a mixture of glucuronides, *N*-acetyltyramine-*O*,*β*-glucuronide (**1**), phenyl-*β*-glucuronide (**2**), *p*-nitrophenyl-*β*–D-glucuronide (**3**), estrone-*β*-D-glucuronide (**4**), 4-methylumbelliferyl-*β*-D-glucuronide (**5**), and *p*-acetamidophenyl-*β*-D-glucuronide (**6**) after treatment with A) 100 U BGTurbo® in solution and B) 100 U BGTurbo® coupled to magnetic beads. The experiments were performed in triplicate (error bars: SD).

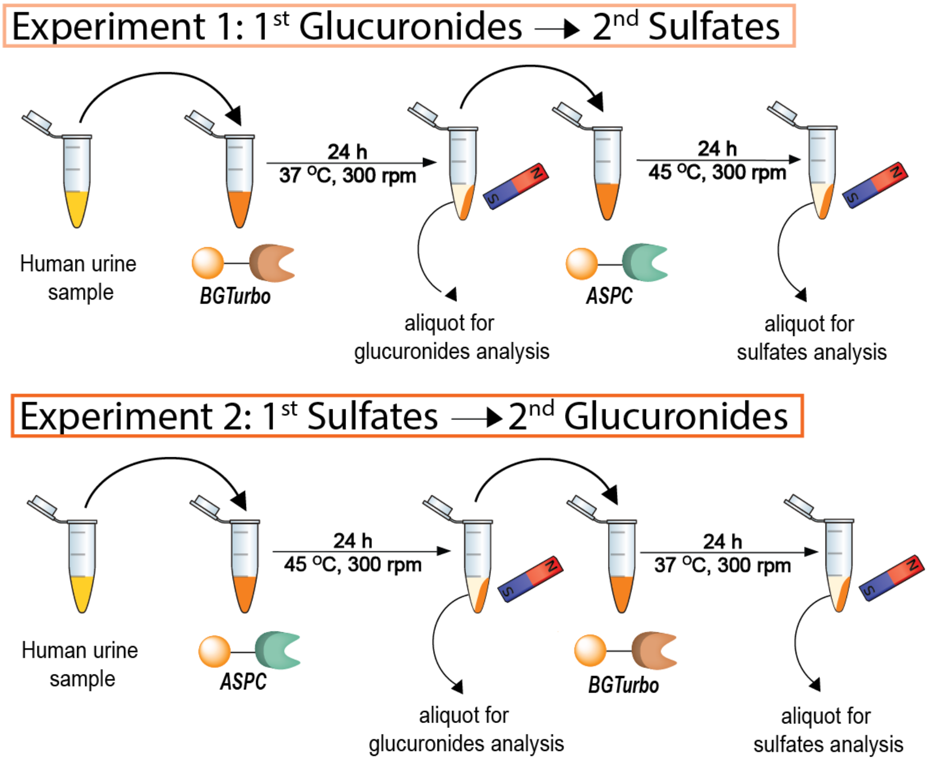

Figure S3. Workflow of our initial experimental design with two cycles. In the first experiment (or cycle), the urine sample is treated first with the BGTurbo® followed by treatment with the ASPC™. In the second experiment (or cycle), the same urine sample is first treated with the arylsulfatase and then with the β–glucuronidase.

A)

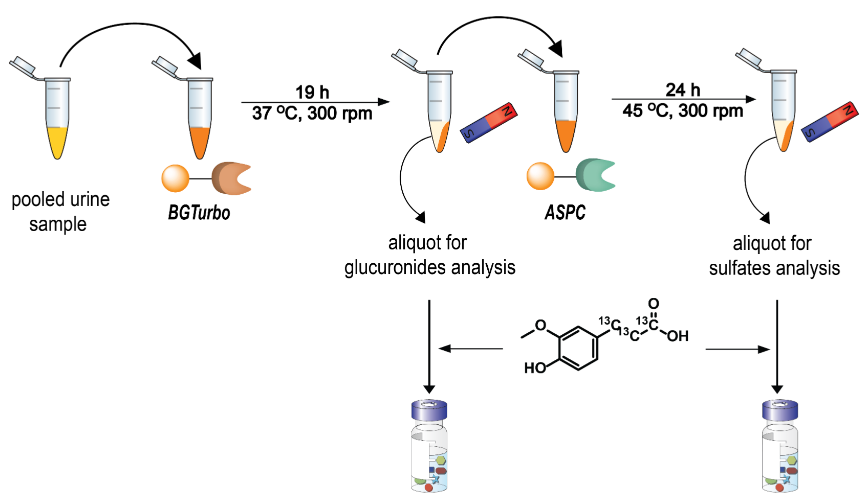

B)

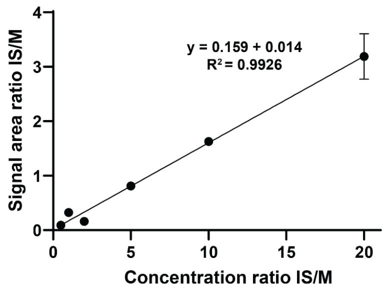

C)

	Concentration (µmol / L)	RSD
Ferulic acid in control	33	19 %
Ferulic acid after glucuronidase treatment	82	8.9 %
Ferulic acid after sulfatase treatment	77	5.6 %

Figure S4. A) Workflow for the quantification of ferulic acid. B) Calibration curve. C) Quantification results. The concentration is normalized to creatinine levels. Data are presented as mean ± SD from experimental replicates (N = 3)

Tables

Table S1. Creatinine concentrations in spot urine samples of volunteers before (V1) and 24 h collection of urine samples after consumption of a (poly)phenol rich breakfast (V2) for 3 days. These concentrations were used for normalization of the pooled urine samples utilized in the serial enzymatic treatment.

Sample	Creatinine concentration (mmol/L)	Sample	Creatinine concentration (mmol/L)
3_V1	1.36	3_V2	2.55
4_V1	4.82	4_V2	4.33
8_V1	4.30	7_V2	8.34
18_V1	22.6	8_V2	6.77
19_V1	8.66	10_V2	4.80
32_V1	16.1	18_V2	2.25
33_V1	8.43	31_V2	5.56
34_V1	7.52	33_V2	2.01
36_V1	3.26	34_V2	4.77
39_V1	6.36	36_V2	3.99

Table S2. Results from metabolite corona formation experiment.

**Glucuronides**	
**Metabolite**	**Recovery**
p-nitrophenyl-*β*-D-glucuronide	75 %
4-methylumbelliferyl-*β*-D-glucuronide	87 %
**Sulfates**	
**Metabolite**	**Recovery**
estrone-3-sulfate	83 %
4-methylumbelliferyl sulfate	73 %
4-nitrocatechol sulfate	80 %

Table S3. Creatinine concentrations in spot urine samples of volunteers before (V1) and 24 h collection of urine samples after consumption of a (poly)phenol rich breakfast (V2) for 3 days. These concentrations were used for normalization of the pooled urine sample analyzed in the quantification experiment of ferulic acid.

Sample	Creatinine concentration (mmol/L)
19_V1	8.66
33_V1	8.43
7_V2	8.34
13_V2	8.44
38_V2	8.10

Table S4. All validated sulfated metabolites with annotated confidence levels.

(Level 1: Validation with authentic synthetic or commercial standards; Level 2a: Metabolite structure validation based on unambiguous matching of MS^2 spectra with experimental spectra from literature or library sources; Level 2b: Identification of the molecular formula and MS^2 fragmentation pattern comparison using computational tools; Level 3: MS^2-validation of sulfate ester moiety in the metabolite).

#	Name	Structure	Chemical formula	m/z Experimental	m/z Theoretical	ppm difference	Level of confidence
1	Phenol sulfate	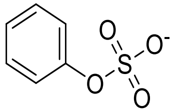	$C_6H_5O_4S^-$	172.9914	172.9914	0.00	1
2	*p*-Cresol sulfate	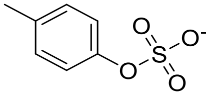	$C_7H_7O_4S^-$	187.0071	187.0071	0.00	1
3	Resorcinol sulfate	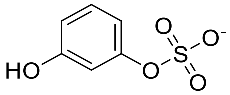	$C_6H_5O_5S^-$	188.9864	188.9863	0.53	2b
4			$C_6H_5O_5S^-$	188.9864			3
5			$C_6H_7O_5S^-$	191.0020			2b
6			$C_7H_5O_5S^-$	200.9864			2b
7	4-Ethylphenylsulfate	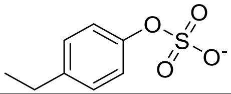	$C_8H_9O_4S^-$	201.0227	201.0227	0.00	2b
8	3-Hydroxy-5-methylphenyl sulfate	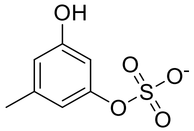	$C_7H_7O_5S^-$	203.0020	203.0020	0.00	2b
9							2b
10			$C_7H_7O_5S^-$	203.0020			2b
11							2b
12				204.9813		0.49	2b
13	Pyrogallol sulfate		$C_6H_5O_6S^-$	204.9812	204.9812	0.00	2b
14				204.9813		0.49	2b

15		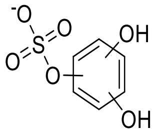		204.9813		0.49	3
16			$C_6H_7O_6S^-$	206.9970			2b
17	Indoxyl sulfate	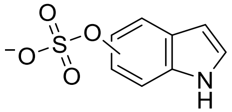	$C_8H_7NO_4S^-$	212.0025	212.0023	0.94	2b
18				212.0024		0.47	2b
19			$C_9H_9O_4S^-$	213.0227	213.0227	0.00	2b
20			$C_8H_7O_5S^-$	215.0024	215.0020	1.86	2b
21	4-Hydroxybenzoic acid sulfate / 2-Hydroxybenzoic acid sulfate	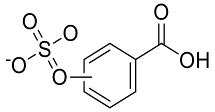	$C_7H_5O_6S^-$	216.9813	216.9812	0.46	2b
22	3-Hydroxybenzoic acid sulfate	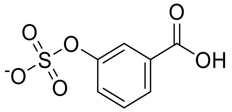	$C_7H_5O_6S^-$	216.9813	216.9812	0.46	1
23	Tyrosol 4-sulfate	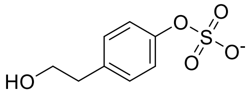	$C_8H_9O_5S^-$	217.0176	217.0176	0.00	2b
24			$C_8H_9O_5S^-$	217.0176			2b
25			$C_7H_7O_6S^-$	218.9970			2b
26			$C_7H_7O_6S^-$	218.9969			2b
27			$C_6H_5O_7S^-$	220.9763			3
28			$C_{10}H_{11}O_4S^-$	227.0384			2b
29			$C_8H_6NO_5S^-$	227.9973			2b
30				227.9971			2b
31			$C_9H_9NO_5S^-$	229.0177			2b
32			$C_{10}H_{13}O_4S^-$	229.0540			2b
33	Paracetamol sulfate	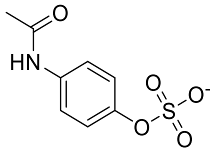	$C_8H_8NO_5S^-$	230.0130	230.0129	0.44	2b

34				230.0129			2b
35			$C_8H_7O_6S$	230.9970			2b
36	2-(4-Methoxyphenyl)ethyl hydrogen sulfate	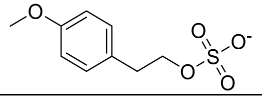	$C_9H_{11}O_5S^-$	231.0332	231.0333	-0.43	2b
37	Dopamine-3-sulfate/ Dopamine-4-sulfate	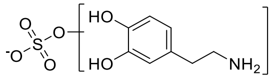	$C_8H_{10}NO_5S^-$	232.0286	232.0285	0.43	2b
38	Hydroxytyrosol sulfate	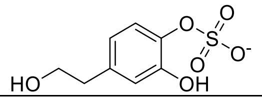	$C_8H_9O_6S^-$	233.0125	233.0125	0.00	2b
39				233.0127		0.86	2b
40	trans-4-Hydroxycinnamic acid sulfate	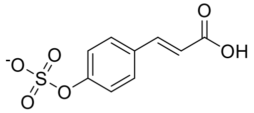	$C_9H_7O_6S^-$	242.9968	242.9969	-0.41	2b
41			$C_{10}H_{11}O_5S^-$	243.0332			2b
42	Phloretic acid sulfate	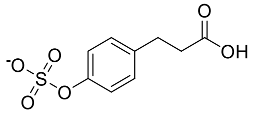	$C_9H_9O_6S^-$	245.0125	245.0125	0.00	2b
43			$C_{10}H_{13}O_5S^-$	245.0490			2b
44	Vanillic acid sulfate	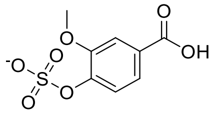	$C_8H_7O_7S^-$	246.9919			1
45	Homovanillyl alcohol sulfate	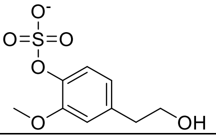	$C_9H_{11}O_6S^-$	247.0283	247.0282	0.41	2b
46			$C_9H_7O_7S^-$	258.9918			2b
47				258.9920			3
48	L-Tyrosine-*O*-sulfate	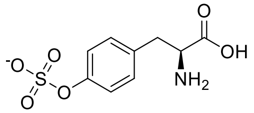	$C_9H_{10}NO_6S^-$	260.0234	260.0234	0.00	2b

49	Dihydrocaffeic acid 3-sulfate	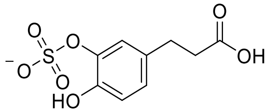	$C_9H_9O_7S^-$	261.0076	261.0074	0.77	2b
50				261.0439			3
51				261.0438			3
52			$C_8H_7O_8S^-$	262.9866			2b
53			$C_8H_7O_6S_2^-$	262.9690			2b
54			$C_7H_7O_7S_2^-$	266.9640			2b
55			$C_{11}H_{11}O_6S^-$	271.0283			2b
56	Ferulic acid 4-*O*-sulfate	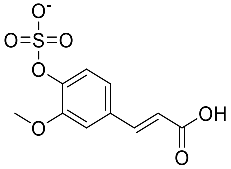	$C_{10}H_9O_7S^-$	273.0074	273.0074	0.00	2a
57			$C_{10}H_{12}NO_6S^-$	274.0391			2b
58	Syringic acid-4-sulfate	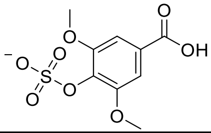	$C_9H_9O_8S^-$	277.0025	277.0018	2.53	2b
59			$C_{11}H_{13}O_8S^-$	287.0232			2b
60			$C_8H_8NO_8S^-$	291.0178			3
61	Sinapic acid sulfate	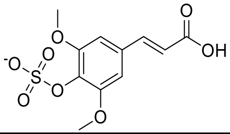	$C_{11}H_{11}O_8S^-$	303.0181	303.0180	0.33	2b
62			$C_{11}H_{13}O_8S^-$	305.0337	305.0337	0.00	3
63	4-hydroxy-5-(4'-hydroxyphenyl)valeric acid-3'-sulfate	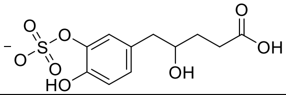		305.0336		-0.33	2b
64			$C_{16}H_{18}NO_6S^-$	352.0858			2b

Table S5. All validated glucuronidated metabolites with annotated confidence levels.

(Level 1: Validation with authentic synthetic or commercial standards; Level 2a: Metabolite structure validation based on unambiguous matching of MS^2 spectra with experimental spectra from literature or library sources; Level 2b: Identification of the molecular formula and MS^2 fragmentation pattern comparison using computational tools; Level 3: MS^2-validation of glucuronic acid moiety in the metabolite).* Gluc: glucuronic acid

				m/z			
#	Name	Structure*	Chemical formula	Experimental	Theoretical	ppm difference	Level of confidence
1			$C_{10}H_{15}O_9^-$	279.0721			2b
2	*p*-Cresol glucuronide	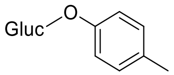	$C_{13}H_{15}O_7^-$	283.0825	283.0818	2.47	1
3	Quinol glucuronide	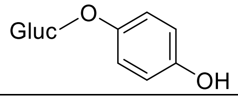	$C_{12}H_{13}O_8^-$	285.0617	285.0611	2.11	2b
4	4-Ethylphenol glucuronide	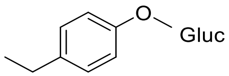	$C_{14}H_{17}O_7^-$	297.0980	297.0980	0.00	2b
5	4-Hydroxybenzyl alcohol glucuronide	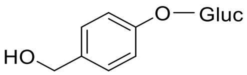	$C_{13}H_{15}O_8^-$	299.0774	299.0772	0.67	2b
6	Pyrogallol-2-O-glucuronide	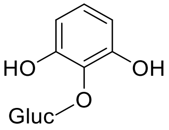	$C_{12}H_{13}O_9^-$	301.0538	301.0565		2b
7			$C_{14}H_{14}NO_7^-$	308.0777			3
8			$C_{14}H_{14}NO_8^-$	324.0725			2b
9				324.0727			2b
10	Acetaminophen glucuronide	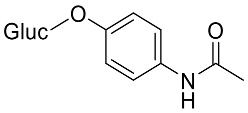	$C_{14}H_{16}NO_8^-$	326.0882	326.0881	0.31	2b
11			$C_{15}H_{14}NO_8^-$	336.0726			2b
12			$C_{16}H_{17}O_8^-$	337.0929			2b

13			$C_{16}H_{19}O_8^-$	339.1087			2b
14	3-(4-Methoxyphenyl)-1-propanol glucuronide	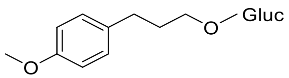	$C_{16}H_{21}O_8^-$	341.1244	341.1242	0.59	2b
15			$C_{14}H_{17}O_{10}^-$	343.0671			2b
16							2b
17			$C_{16}H_{23}O_8^-$	343.1399			2b
18			$C_{16}H_{16}NO_8^-$	350.0882			2b
19			$C_{12}H_{16}O_{12}^-$	351.0571			
20			$C_{12}H_{20}O_{12}^-$	355.0896			2b
21			$C_{16}H_{19}O_9^-$	355.1036			2b
22	Homovanillic acid 4-glucuronide	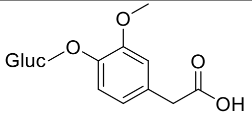	$C_{15}H_{17}O_{10}^-$	357.0826	357.0827	-0.28	2a
23	Dihydrocaffeic acid 3-O-glucuronide	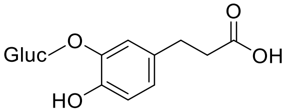		357.0829		0.56	2b
24	Hydroxyphenyllactic acid glucuronide	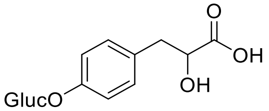		357.0829		0.56	2a
25			$C_{16}H_{25}O_9^-$	361.1507			2b
27				361.1505			2b
28			$C_{17}H_{19}O_9^-$	367.1036			2b
29	trans-Ferulic acid 4-glucuronide	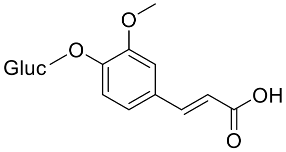	$C_{16}H_{17}O_{10}^-$	369.0829	369.0827	0.54	2b
30	Dihydroisoferulic acid 3-glucuronide	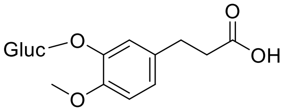	$C_{16}H_{19}O_{10}^-$	371.0985	371.0984	0.27	2b
31			$C_{16}H_{21}O_{10}^-$	373.1141			2b
32			$C_{16}H_{22}O_{10}^-$	375.1297			3

33				377.1495			3
34			$C_{16}H_{27}O_{10}^{-}$	379.1611			3
35			$C_{12}H_{13}O_{12}S^{-}$	381.0135			3
36				381.1014			3
37				387.1663			3
38				387.2025			3
39			$C_{18}H_{28}O_{9}^{-}$	389.1818			3
40				395.0293			3
41			$C_{17}H_{21}O_{11}^{-}$	401.1090			2b
42	Urolithin A-8-O-glucuronide	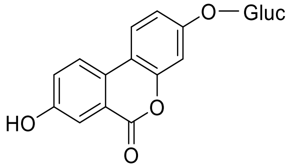	$C_{19}H_{17}O_{10}^{-}$	403.0673	403.0671	0.50	2b
43				415.1974			3
44	Daidzein 4'-O-glucuronide	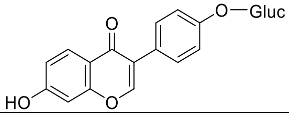	$C_{21}H_{17}O_{10}^{-}$	429.0829	429.0827	0.47	2b
45				429.1769			3
46			$C_{21}H_{21}O_{10}^{-}$	433.1141	433.1140	0.23	2b
47			$C_{20}H_{27}O_{11}^{-}$	443.1560			2b
48	Apigenin 7-glucuronide	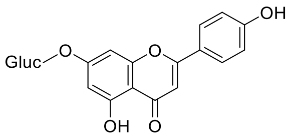	$C_{21}H_{17}O_{11}^{-}$	445.0777	445.0776	0.23	2b
49			$C_{24}H_{25}O_{10}^{-}$	473.1456			3
50			$C_{24}H_{29}O_{10}^{-}$	477.1767			2b
51			$C_{25}H_{37}O_{9}^{-}$	481.2444			2b
52			$C_{26}H_{40}O_{10}^{-}$	511.2550			2b
52			$C_{24}H_{30}O_{13}S$	557.1335			2b

General methods

All reagents and solvents were purchased from Sigma-Aldrich or Fischer Scientific and were used without further purification. HPLC grade solvents were used for HPLC purification and mass spectrometry grade for UHPLC-ESI-MS analysis. All biochemical reactions were performed with HPLC or LC-MS grade solvents. Solutions were concentrated in vacuo on a Speedvac Concentrator Plus System (Eppendorf, Hamburg, Germany). High-resolution mass spectra were acquired on a Maxis II ETD Q-TOF mass spectrometer (Bruker Daltonics, Germany) using an electrospray ionization (ESI) source with an Elute UHPLC (Bruker Daltonics, Germany) or 1260 Infinity II Binary Pump (Agilent Technologies, USA) system and equipped with a Waters ACQUITY UPLC HSS T3 column (1.8 mm, 100x2.1 mm).

Study design

An open-label, single-arm study investigating the variability in polyphenol gut microbial metabolism and the impact on vascular response in healthy males and females is currently ongoing. The final cohort has been powered accordingly and expects to recruit a total population of 250 volunteers. In the present work, we analyze a sub-sample of a total of 22 volunteers, recruited from King's College London and surrounding areas. Inclusion criteria comprised good general health, age between 20 and 70 years old, and BMI range between 18.5 and 35 kg/m^2. Exclusion criteria included history of cardiovascular disease, hypertension, history of diabetes, metabolic syndrome, terminal renal failure or malignancies, abnormal heart rhythm (below 60 or above 100 bpm), allergy to berries, flaxseed or soy, smoke an irregular number of cigarettes, taking medications that can affect the cardiovascular system, recent loss of more than 10% of weight, pregnancy or planning to become pregnant in the next 6 months, or participation in another study in the past month. Participants were asked to refrain from vegetables, fruits, wine, cocoa, chocolate, tea and coffee 24 h prior to the first visit to reduce the influence of background diet. All subjects gave written informed consent before their participation in the study and agreed to maintain their eating/drinking and exercise habits for the duration of the study.

Subjects consumed a (poly)phenol breakfast containing 30 g of milled flaxseeds (containing 300 mg of lignans), 40 g of freeze-dried raspberry powder (containing 153 mg of ellagitannins) and 250 mL of soy milk (containing 22 mg of isoflavones) for 3 days.[1] Spot urine samples were collected in a fasted state on day 1 and a 24 h urine sample was collected after consumption of the last breakfast on day 3. Volume was recorded and samples were stored at

−80 °C. The study was conducted in accordance to the guidelines stated in the current revision of the Declaration of Helsinki, and informed consent was obtained for all subjects. All procedures involving human subjects were approved by King's College London Research Ethics Committee (HR-17/18–5353) and registered at the National Institutes of Health clinicaltrials.gov as NCT03573414.

Description of procedures

Determination of glucuronidase activity

Glucuronidase activity was tested according to the protocol described by Sigma Aldrich (S9626). In order to calculate the activity of glucuronidase in solution, 65 µL of H_2O were mixed with 50 µL of 75 mM potassium phosphate buffer with 1% (w/v) bovine serum albumin, pH 6.8, 25 µL of 3 mM of phenolphthalein-glucuronide and 10 µL of enzyme test solution. A negative control was also tested, in which no enzyme was added. To stop the reaction, 500 µL of 200 mM glycine buffer, pH 10.4 were added. The resulting solution was transferred to a 96-well plate and the absorbance at 540 nM was measured to monitor the production of phenolphthalein.

At the same time, a phenolphthalein standard curved was prepared, with a ranging quantity of 1-5 µg. The amount of phenolphthalein was plotted against the A540 value and test results were based on the measured absorbance.

The amount of units in solution was calculated using the following equation:

$$Units/mL = \frac{(\mu\text{g of phenolphthalein released}) \times \text{df}}{\text{V}_\text{E}\ x\ t}$$

Details:

t – Time factor correction (Unit definition for 1 hour)

df – Protein dilution factor

V_E – Volume (in mL) of purified glucuronidase used

Determination of arylsulfatase activity

The arylsulfatase activity assay was based on the assay described for the *Helix pomatia* arylsulfatase (S9626, Sigma-Aldrich). Briefly, for each enzymatic assay, 65 µL of 200 mM sodium acetate buffer pH 5 and 40 µL of a 6.25 mM aqueous solution of 4-nitrocatechol sulfate were mixed. To this mixture were added 5, 7 or 10 µL of 50 times diluted purified arylsulfatase. At the same time, an assay was performed without any enzyme, as a negative control. The mixtures were incubated for 30 min at 37 °C. After incubation, 500 µL of 1 M NaOH were added to the reaction and the resulting solutions were transferred into a 96-well plate. Their absorbance was measured at a wavelength of 515 nm.

The amount of units in solution was calculated using the following equation:

$$Units/mL = \frac{(\mathrm{A_{Test}} - \mathrm{A_{Blank}}) \times \mathrm{df} \times \mathrm{V_T}}{\varepsilon_{515}\ x\ \mathrm{V_E}\ x\ t}$$

Details:

A_{Test} – Absorbance measured for the test solutions at 515 nm

A_{Blank} – Absorbance measured for the blank at 515 nm

t – Time factor correction (Unit definition for 1 hour)

df – Protein dilution factor

V_T – Total volume (in mL) of the assay

ε_{515} – Milimolar extinction coefficient of *p*-nitrocatechol at 515 nm ($\mu m^{-1} cm^{-1}$)

V_E – Volume (in mL) of purified arylsulfatase used

Carry-over effect

100 U of the *β*-glucuronidase BGTurbo® were immobilized to the magnetic beads. Six cycles of 1 h incubation were performed using 5 different substrates, *N*-acetyltyramine-*O*,*β*–glucuronide (NATOG), *p*-nitrophenyl-*β*-D-glucuronide (*p*-NPG), *p*-acetamidophenyl-*β*-D-glucuronide (*p*-APG), 8-hydroxyquinoline-*β*-D-glucuronide (8-HQG), 4-methylumbelliferyl-*β*-D-glucuronide (MUG). The concentration was 591 µM for NATOG and 500 µM for the rest of the glucuronides. The

percentages of the carry-over within each cycle were calculated for both the glucuronides and the aglycons in all samples (control and treated) using the following formula:

% carry-over = (post-cycle analyte peak area) / (cycle analyte peak area) * 0.01

example: % carry-over (NATOG) = (peak area of NATOG in the 2nd cycle) / (peak area of NATOG in the 1st cycle) * 0.01

Cycle analyses of the immobilized enzymes

The reusability of the immobilized enzymes to magnetic beads was examined in two individual experiments for the immobilized β-glucuronidase and arylsulfatase by performing 7 cycles of 1 h incubation. For the immobilization of BGTurbo® and ASPC™, 100 µL of MagnaBind Carboxyl Derivatized Beads slurry was used (Protocol described in **Immobilization to magnetic beads**). For the control assay no enzyme was added to the magnetic beads.

For the β-glucuronidase treatment, 80 U of BGTurbo® were immobilized to magnetic beads, then 25 µL of 591 µM NATOG were added together with 565 µL Instant Buffer I (Kura Biotech, LOT No: 2319) and incubated for 1 h at 37 °C, 300 rpm, in a Thermomixer. After 1 h an aliquot was removed from both the control and the enzymatic assay for the analysis. Then the magnetic beads were washed twice with 100 µL 25 mM MES buffer for 5 min with thorough shaking (Thermomixer, 25 °C, 400 rpm). The same process was repeated for the next 6 cycles. For the arylsulfatase treatment, 0.1 U of ASPC™ were immobilized to magnetic beads, then 25 µL of 500 µM 4-nitrophenyl sulfate (4-NPS) were added together with 165 µL Instant Buffer II (Kura Biotech, LOT No: 2506) and incubated for 1 h at 45 °C, 300 rpm, in a Thermomixer. After 1 h an aliquot was removed both from the control (beads with no immobilized enzyme) and the enzymatic assay for the analysis. Then the magnetic beads were washed twice with 100 µL 25 mM MES buffer for 5 min with thorough shaking (Thermomixer, 25 °C, 400 rpm). The same process was repeated for the next 6 cycles.

For both enzymatic treatment assays, 100 µL of LC-MS grade MeOH were used to quench the aliquots that were removed for the analysis. The solution was mixed and left on ice for 15 minutes. Then, followed centrifuging for 5 minutes at 14,100 g and the supernatant was transferred into new labeled Eppendorf tubes (carefully to not disrupt the pellet). The samples were dried on SpeedVac (2.0 – 3.0 h) and finally resuspend in 50 µL LC-MS grade ACN 5%. They were transferred into labeled LC-MS vials and injected in a randomized list.

Metabolite corona formation

A mixture of two glucuronides (*p*-nitrophenyl-*β*-D-glucuronide, 4-methylumbelliferyl-*β*-D-glucuronide) and three sulfates (estrone-3-sulfate, 4-methylumbelliferyl sulfate, 4-nitrocatechol sulfate) was prepared at a concentration of 500 µM in Milli-Q water. 200 µL of MagnaBind Carboxyl Derivatized Beads were divided into two different eppendorfs (100 µL for incubation of glucuronides and 100 µL for incubation of sulfates). The incubation for the glucuronides was performed for 19 h at 37 °C, 300 rpm, in a Thermomixer and the incubation for the sulfates was performed for 24 h at 45 °C, 300 rpm, in a Thermomixer. A control sample containing the mixture of the compounds and no magnetic beads was incubated under the same conditions. After each incubation, 50 µL were removed, 2 µL of $^{13}C_9$ L-phenylalanine was spiked as internal standard (IS) and the samples were injected in the UHPLC-MS.

The recovery was calculated after normalization to the IS (normalized analyte peak area) towards the control sample as follows:

$$\% \text{ recovery} = (\text{normalized analyte peak area})_{\text{beads}} / (\text{normalized analyte peak area})_{\text{mixture}} * 0.01$$

Two-cycles experiment

400 µL of pooled human urine was divided into two different eppendorfs (200 µL for BGTurbo® assay: 100 µL for control and 100 µL for enzymatic treatment and 200 µL for ASPC™ assay: 100 µL for control and 100 µL for enzymatic treatment) and precipitated with four times cold LCMS-grade MeOH for 30 min at 0 °C. After centrifugation (14,100 g, 5 min), the supernatant was removed and concentrated in speedvac. The pellet was then dissolved in the following volumes: for BGTurbo® vial: 200 µL Instant Buffer I and for ASPC™ vial: 200 µL Instant Buffer II.

For the BGTurbo® treatment, from the 200 µL of reconstituted sample, 100 µL were added into the control assay and the other 100 µL were added to 100 U of BGTurbo® immobilized to magnetic beads (Protocol described in **Immobilization to magnetic beads**) and incubated overnight at 37 °C, 300 rpm, in a Thermomixer. Aliquots were removed at 0 min for the control assay and at 24 h for control and enzymatic assays. For the ASPC™ treatment, from the 200 µL of reconstituted sample, 100 µL were added into the control assay and the other 100 µL were added to 5 U of ASPC™ immobilized to magnetic beads (Protocol described in **Immobilization to magnetic beads**) and incubated overnight at 45 °C, 300 rpm, in a Thermomixer. Aliquots were removed at 0 min for the control assay and at 24 h for control and enzymatic assays.

For both enzymatic treatment assays, 100 µL of LC-MS grade MeOH were used to quench. The solution was mixed and left on ice for 15 minutes. Then, followed centrifuging for 5 minutes at 14,100 g and the supernatant was transferred into new labeled Eppendorf tubes (carefully to not disrupt the pellet). The samples were dried on SpeedVac (2.0 – 3.0 h) and finally resuspend in 50 µL LC-MS grade ACN 5%. They were transferred into labeled LC-MS vials and injected 6 times each in a randomized list.

Quantification of ferulic acid

Isotope-labeled Ferulic acid-1,2,3-$^{13}C_3$ was purchased from Sigma-Aldrich. A total of 600 µL of pooled urine samples was used for the quantification experiment. Ice cold methanol (2.4 mL) was added to the pooled urine sample for protein precipitation. The sample was vigorously shaken for 30 s and then cooled at 4 °C for 30 min. Upon protein precipitation and centrifugation at 14,100 g for 5 min, equal amounts of the supernatant containing the extracted urine metabolite mixture was transferred into six separate tubes (three replicates for control and three replicates for the enzymatic treatment assays) and dried under vacuum in a Speedvac. The residues of the tubes were dissolved in 100 µL of Instant Buffer I. For the immobilization of BGTurbo® and ASPC™, 100 µL of MagnaBind Carboxyl Derivatized Beads slurry was used (Protocol described in **Immobilization to magnetic beads**). For the control assay no enzyme was added to the magnetic beads.

After the 19 h incubation with BGTurbo®, the beads were placed on the magnet and 25 µL of the solution were removed for the analysis of the glucuronidated metabolites. The remaining supernatant was transferred for the sulfatase treatment to the beads coupled to ASPC™ and after 24 h of incubation the beads were placed on the magnet and 25 µL of the solution were removed for the analysis of the sulfated metabolites. Ice cold methanol (100 µL) was added to the 25 µL aliquots and $^{13}C_3$-Ferulic acid (5 µL, 100 nM) was spiked into the solutions. The mixtures were then dried under vacuum and redissolved in 50 µL LC-MS grade ACN 5% before submission to the UPLC-MS analysis. The quantification of ferulic acid was repeated three times for each 100 µL urine samples.

Reference

1. M. S. P. Correia, A. Jain, W. Alotaibi, P. Young Tie Yang, A. Rodriguez-Mateos and D. Globisch, *Free Radic Biol Med* 2020, **160**, 745-754.
